# Supplementary material for: Crossed laser phase plates for transmission electron microscopy
Source: Nat Commun. 2026 Jun 5;17:7199. doi: 10.1038/s41467-026-74060-6 (PMC13396383; doi:10.1038/s41467-026-74060-6)
Supplement: Supplementary file 1 — Supplementary Information [file 41467_2026_74060_MOESM1_ESM.pdf]

Supplementary Materials for "Crossed laser phase plates for transmission electron microscopy"

Petar N. Petrov[1,2], Jessie T. Zhang[1,2], Jeremy J. Axelrod[†1], Pavel K. Olshin[3], and Holger Müller[1,2,*]

[1]Department of Physics, University of California, Berkeley, Berkeley, CA 94720, USA
[2]Lawrence Berkeley National Laboratory, One Cyclotron Road, Berkeley, CA 94720, USA
[3]Biohub, Redwood City, CA 94063, USA
[*]Corresponding author: hm@berkeley.edu

Supplementary Note 1: Derivation of the relativistic XLPP phase shift

In the non-relativistic limit, the interaction between the electromagnetic field and the electron beam is given by the ponderomotive potential, which does not depend on the polarization of the electromagnetic field and repels the electrons from the high-intensity antinodes of the laser standing wave. For relativistic electron beams, as found in practice, the interaction becomes polarization-dependent. For electrons moving at velocities v_e larger than $c/\sqrt{2}$ (electron energies larger than $\sim$211 keV), the interaction can even become reversed, such that the antinodes appear attractive to the electron beam while the nodes become repulsive [1]. In this section, we will derive the relativistic phase shift profile created by the XLPP.

In the laboratory frame in Coulomb gauge, the phase shift η imparted to the electron matter waves by the laser is [1]

$$\eta = \frac{\mathrm{e}^2}{2m\hbar}\int \frac{dt}{\gamma}\left[\left(\mathbf{A}(\mathbf{d}_0(t),t) - \nabla G(\mathbf{d}_0(t),t)\right)^2 - \beta^2\left(A_z(\mathbf{d}_0(t),t) - \nabla_z G(\mathbf{d}_0(t),t)\right)^2\right], \tag{1}$$

with $-\mathrm{e}, m$ the electron charge and mass, $\mathbf{A}$ the vector potential of the laser field with $A_z := \mathbf{A}\cdot\hat{\mathbf{r}}_z$, $\mathbf{d}_0(t)$ the unperturbed electron trajectory, G a gauge function that arises from the Lorentz transformation between the lab frame and the frame in which the uperturbed electron beam is at rest, $\beta = v_e/c$ the normalized electron velocity, and $\gamma = 1/\sqrt{1-\beta^2}$. The vector potential $\mathbf{A}(\mathbf{r},t) = \mathbf{A}_1(\mathbf{r},t) + \mathbf{A}_2(\mathbf{r},t)$ of the XLPP is given by adding two standing waves propagating in the $\hat{\mathbf{r}}_x$- and $\hat{\mathbf{r}}_y$-direction, respectively:

$$\mathbf{A}_1(\mathbf{r},t) = A_1(y,z)\cos(kx)\left[\cos(\theta_1)\cos(\omega t)\hat{\mathbf{r}}_z + \sin(\theta_1)\cos(\omega t - \varepsilon_1)\hat{\mathbf{r}}_y\right], \tag{2}$$

$$\mathbf{A}_2(\mathbf{r},t) = A_2(x,z)\cos(ky)\left[\cos(\theta_2)\cos(\omega t + \Omega)\hat{\mathbf{r}}_z + \sin(\theta_2)\cos(\omega t - \varepsilon_2 + \Omega)\hat{\mathbf{r}}_x\right] \tag{3}$$

where $\mathbf{r} := (r_x, r_y, r_z)$ are the spatial coordinates, θ_j are polarization angles relative to the $\hat{\mathbf{r}}_z$ axis, ε_j are ellipticity parameters, $k = 2\pi/\lambda_l$ is the angular wave number, and Ω is the temporal phase of the two standing waves. Assuming the envelope functions $A_{1,2}$ are slowly-varying relative to the wave cycle along the electron trajectory [1], we can time-average the integrand of Supplementary Equation (1) over one period $T = 2\pi/\omega$ of the optical field, writing η in terms of an effective potential U,

$$\eta = \frac{1}{\hbar}\int dt\, U(\mathbf{r}), \tag{4}$$

$$U(\mathbf{r}) = \frac{\mathrm{e}^2}{2m\gamma}\frac{1}{T}\int_0^T dt\, \left[\left(\mathbf{A}(\mathbf{r},t) - \nabla G(\mathbf{r},t)\right)^2 - \beta^2\left(A_z(\mathbf{r},t) - \nabla_z G(\mathbf{r},t)\right)^2\right], \tag{5}$$

where we note that $r_z = c\beta t$. In the slowly-varying envelope approximation, we can also approximate the

gauge function as [1]

$$G(\mathbf{r},t) \approx \frac{c\beta}{\omega}\left[A_1(r_y,r_z)\cos(kr_x)\cos(\theta_1)\sin(\omega t) + A_2(r_x,r_z)\cos(kr_y)\cos(\theta_2)\sin(\omega t+\Omega)\right]. \tag{6}$$

Under these conditions, Supplementary Equation (4) gives the phase shift for an arbitrary configuration of the parameters $\{\theta_1, \theta_2, \varepsilon_1, \varepsilon_2, \Omega\}$.

Special cases

We will consider the three special cases of laser beams polarized vertically, horizontally, and at the "relativistic reversal angle."

Vertical polarization. When the two laser polarizations are vertical ($\theta_{1,2} = 0$), we find

$$U(\mathbf{r}) = U_v(\mathbf{r}) = \frac{\mathrm{e}^2}{4m\gamma}\left\{\frac{A_1^2(r_y,r_z)}{2}\left[1+(1-2\beta^2)\cos(2kr_x)\right] + \frac{A_2^2(r_x,r_z)}{2}\left[1+(1-2\beta^2)\cos(2kr_y)\right]\right.$$
$$\left. + 2A_1(r_y,r_z)A_2(r_x,r_z)\cos(kr_x)\cos(kr_y)\cos(\Omega)(1-\beta^2)\right\}. \tag{7}$$

We note that the terms in A_j^2 have the same form as those in a single phase plate [1], wherein the effective potential appears as a standing wave with its modulation depth scaled by $(1-2\beta^2)$. In the non-relativistic limit $\beta \to 0$, we observe that the potential reduces to

$$U_v(\mathbf{r}) = \frac{\mathrm{e}^2}{4m\gamma}|A_1(r_y,r_z)\cos(kr_x) + A_2(r_x,r_z)\cos(kr_y)e^{i\Omega}|^2, \tag{8}$$

such that the phase shift η is proportional to the integral of the laser intensity along $\hat{\mathbf{r}}_z$. However, we note that at the accelerating voltages used in cryo-EM, the β-dependent terms cannot be neglected. The consequences of this for the CTF are explored in Supplementary Note 2.

Horizontal polarization. When both laser polarizations are horizontal ($\theta_{1,2} = \frac{\pi}{2}$), the two laser beams can no longer interfere and the effective potential is simply the sum of that coming from two SLPPs,

$$U(\mathbf{r}) = U_h(\mathbf{r}) = \frac{\mathrm{e}^2}{4m\gamma}\left\{\frac{A_1^2(r_y,r_z)}{2}\left[1+\cos(2kr_x)\right] + \frac{A_2^2(r_x,r_z)}{2}\left[1+\cos(2kr_y)\right]\right\}. \tag{9}$$

We can see that the resulting potential is similar to that of Supplementary Equation (7) in the limit $\beta \to 1$ except that the modulation depth of the standing waves is inverted in the latter case.

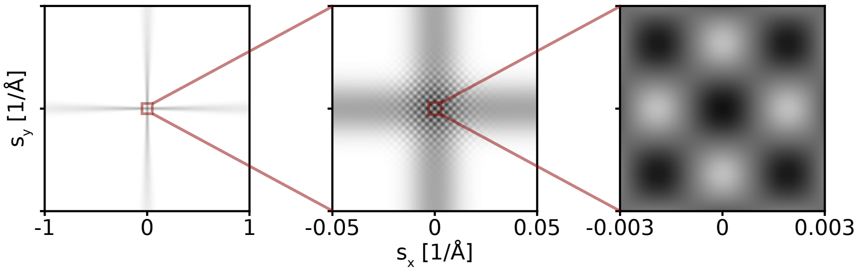

Supplementary Figure 1: **Phase shift at the relativistic reversal angle.** Progressively zoomed-in view of the phase shift produced by a XLPP with both laser beams polarized at the RRA (with $N_A = 0.08$). Middle panel shows the difference between the central region where the beams interfere and the outer regions where the standing wave structure of the laser intensities is washed out by the polarization-dependent relativistic effect. White corresponds to zero phase shift and black to the maximum.

Relativistic reversal angle polarization. When $\beta \geq 1/\sqrt{2}$, the polarization-dependence of the laser-electron interaction permits a unique feature of η. At the so-called relativistic reversal angle (RRA), $\theta_r := \arccos(1/\sqrt{2}\beta)$, the nodes and antinodes of a laser standing wave create the same phase shift [1]. In this case, the potential of the XLPP simplifies to

$$U(\mathbf{r}) = U_r(\mathbf{r}) = \frac{\mathrm{e}^2}{4m\gamma}\left\{\frac{1}{2}\left[A_1(r_y, r_z)^2 + A_2(r_x, r_z)^2\right] + \frac{1-\beta^2}{\beta^2} A_1(r_y, r_z) A_2(r_x, r_z) \cos(\Omega) \cos(kx) \cos(ky)\right\}. \tag{10}$$

The phase shift away from the origin takes on a smooth Gaussian shape, while near the origin the pattern is complicated by the interference of the two laser beams. The phase pattern that results is plotted in Supplementary Figure 1.

Supplementary Note 2: Effects of laser polarization

As shown in Supplementary Note 1, the phase shift η produced by the XLPP depends significantly on the polarization of the laser beams. In this section, we consider the effects of laser polarization on the CTF by examining the three special cases derived above: when the lasers are polarized vertically, horizontally, and at the RRA.

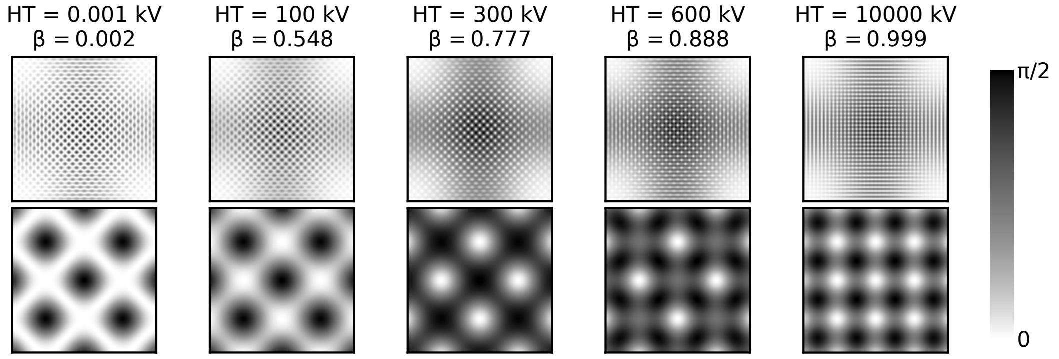

Supplementary Figure 2: **Dependence of phase shift η on electron velocity β in a vertically-polarized XLPP.** Each column shows the phase shift η as experienced by electrons with a different accelerating voltage (HT) through a XLPP with vertically-polarized ($\theta_{1,2} = 0$) lasers. Bottom panel is zoomed in 10× around the center relative to the top panel and in all cases the phase shift is linearly scaled so that the peak phase shift is $\frac{\pi}{2}$ (black). Top panel field of view is 20 µm while bottom panel field of view is 2 µm along each side.

Vertical polarization maximizes the interference of the two lasers. The cross-term produced by laser interference results in a favorable cut-on frequency in the non-relativistic case (Supplementary Equation 8), but examination of the relativistic interaction reveals that this feature gradually disappears and the cut-on frequency gradually increases as the electron velocity β is increased (Supplementary Figure 2). It should be noted that λ_e changes with β, which affects the size of the diffraction pattern.

When the lasers are horizontally-polarized, they cannot interfere, so the cut-on frequency remains fixed as β is varied. For low β, therefore, this configuration produces a worse cut-on frequency than vertical polarization, but as seen in Supplementary Figure 2, the performance of the latter is limited at high β. The transition occurs around an accelerating voltage of ∼100 kV, as shown in Supplementary Figure 3.

When the accelerating voltage exceeds ∼211 kV, operation of the lasers at the RRA is a viable option to eliminate the modulation of η along the laser beams at high spatial frequencies, as seen in Supplementary Figure 1. Relative to horizontal polarization, the ghost image contrast is suppressed by ∼20 % in this configuration, and the smoothing of η at high spatial frequencies suppresses the high-spatial-frequency components in the ghost images (Supplementary Figure 4). However, the cut-on frequency is somewhat worse.

Evidently, vertical polarization is favored at accelerating voltages at or below ∼100 kV for its low cut-on frequency, while horizontal polarization is favorable at higher voltages. At voltages above ∼211 kV, operation

at the RRA may be desirable for its suppression of ghosts. However, the reduction in ghost contrast is modest and the amplitude of high-spatial-frequency components of ghosts is relatively low (see main text Figure 3), so it remains to be seen whether this configuration is worth the considerably more demanding alignment than the horizontal XLPP. Supplementary Figure 5 compares the azimuthally-averaged modulus of the CTF between horizontal and RRA polarizations of the XLPP at the very high N_A of 0.2, demonstrating that, despite a slight increase in cut-on frequency, the latter yields contrast transfer satisfyingly close to the ideal Zernike phase plate.

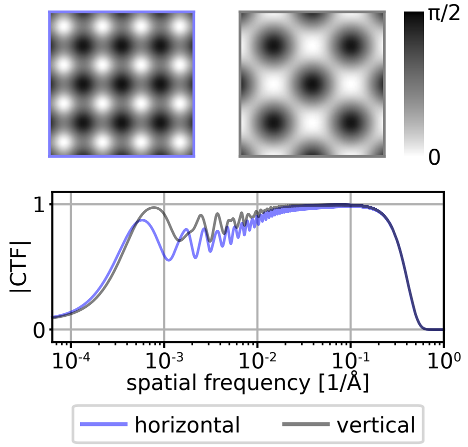

Supplementary Figure 3: **Polarization-dependence of η at 100 kV accelerating voltage.** Top panel shows the zoomed-in phase shift for horizontally-polarized beams (left) and vertically-polarized beams (right), each with a field of view of 2 µm. In both cases, laser power is set so that peak phase shift is $\frac{\pi}{2}$. Bottom panel shows the azimuthally-averaged modulus of the CTF.

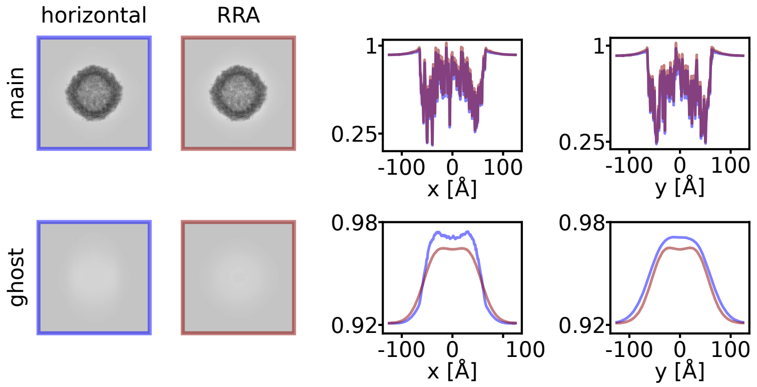

Supplementary Figure 4: **Ghost suppression by relativistic reversal.** Top row shows a main image of an isolated apoferritin particle for a XLPP with $N_A = 0.08$ and either horizontal (blue) or RRA (red) polarization. Horizontal (x) and vertical (y) line scans through the centers of the two images are shown. Bottom row shows the same plots for first-order ghost images. RRA polarization suppresses peak ghost contrast by $\sim$20 %. Horizontal scans show that high-spatial-frequency information in the ghost is lower with RRA polarization than horizontal polarization. Field of view in images is 250 Å along each side.

Supplementary Note 3: Removal of CTF oscillations

As discussed above, the use of a phase plate in cryo-EM obviates the need for defocus and spherical aberration. Operating with a SLPP or XLPP in the "in-focus" condition with spherical aberration correction ($Z = C_s =$

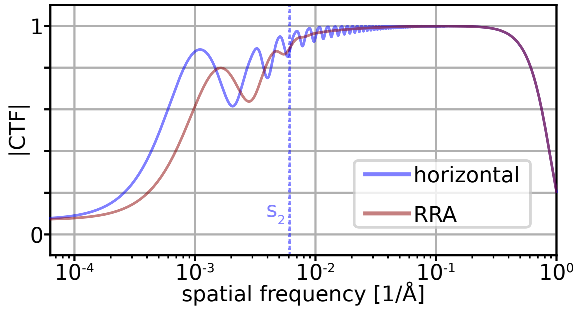

Supplementary Figure 5: **Contrast transfer at N_A of 0.2.** Azimuthally-averaged modulus of the CTF is plotted for horizontal and RRA polarizations of the XLPP. The former has a lower first cut-on frequency but the latter does not have amplitude oscillations above s_2 due to the washing out of the standing wave patterns away from the laser intersection.

0) yields the CTFs shown in Figure 1d (main text), which have no oscillations at high spatial frequencies. This results in a doubling of the spectral power of images at the high spatial frequencies relative to the usual case of $Z \approx -1\,\mu\text{m}$ and $C_s \approx 2.7\,\text{mm}$. For comparison, the latter configuration is illustrated in Supplementary Figure 6, both with and without a LPP. While incorporating a phase plate into this configuration increases the CTF substantially at low spatial frequencies ($|\mathbf{s}| < s_2$), the phase plate alone is not sufficient to eliminate the oscillations of the CTF at high spatial frequencies, which are caused by the terms in χ (main text Equation (6)) which are quadratic (defocus) and quartic (spherical aberration) in $|\mathbf{s}|$.

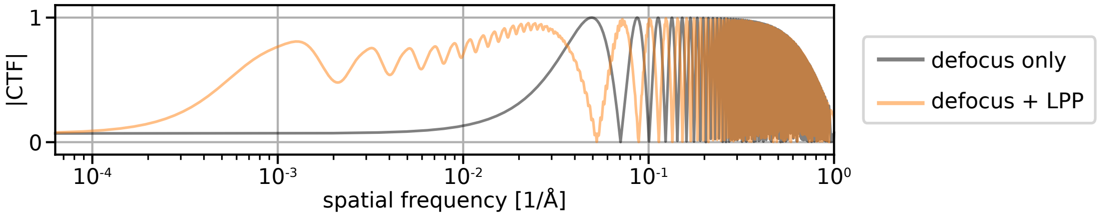

Supplementary Figure 6: **CTF oscillations caused by defocus and spherical aberration.** Azimuthally-averaged modulus of the CTF is plotted for the case of $Z = -1\,\mu\text{m}$ and $C_s = 2.7\,\text{mm}$ in the absence of a phase plate (gray) and when using a SLPP with $N_A = 0.05$ (orange). Additional calculation parameters are provided in Supplementary Table 1.

Supplementary Note 4: Calculation parameters

Supplementary Table 1 lists the typical values of relevant parameters used in the calculations throughout this study. Deviations from these values are specified in the text, figure captions, and/or figure legends.

Parameter	Symbol	Value
effective focal length	f	14.1 mm
energy spread (FWHM)	–	0.3 eV
amplitude contrast	κ	0.07
specimen thickness	–	250 Å
chromatic aberration	C_c	5.1 mm
spherical aberration	C_s	2.7 mm or zero
defocus	Z	−1 µm or zero
electron wavelength	λ_e	1.97 pm
mirror radius of curavture	R	10 mm
laser wavelength	λ_l	1064 nm
laser polarization ellipticities	$\varepsilon_{1,2}$	zero
laser polarization angles	$\theta_{1,2}$	$\pi/2$ rad
laser temporal phase	Ω	zero

Supplementary Table 1: **Values of parameters used in calculations throughout the paper, unless otherwise indicated.** Parameters indicated with – are not assigned a symbol in the text. The non-zero values of Z, C_s are used in the "conventional" configuration in Supplementary Figure 6; elsewhere, the "in-focus" condition is used, with $Z = C_s = 0$.

Supplementary Note 5: Derivation of the CTF

To derive the CTF as used in this paper, we consider image formation in the case of a plane wave of electrons traveling along the optical axis and incident upon a weakly, elastically scattering specimen. The "exit wave" ψ_e may be written

$$\psi_e(\mathbf{x}) = e^{i\varphi(\mathbf{x})}e^{-\mu(\mathbf{x})} = 1 + \psi_s(\mathbf{x}) \tag{11}$$

where $\mathbf{x} = (x, y)$ denotes the spatial coordinates in the object (specimen) plane of the microscope and ψ_s denotes the "scattered" component of the electron wave function. The microscope is described by the transfer function $H(\mathbf{s}) = E(\mathbf{s}) \cdot e^{-i\chi(\mathbf{s})}$, where $E(\mathbf{s})$ describes the coherence envelope and $\chi(\mathbf{s})$ is the phase aberration in Equation (6) in the main text. The image formed on the detector is given by

$$|\psi_i(\mathbf{x})|^2 = |\mathcal{F}^{-1}[\mathcal{F}[\psi_e] \cdot H](\mathbf{x})|^2 \tag{12}$$

$$= |H(\mathbf{0}) + \left(\psi_s * \mathcal{F}^{-1}[H]\right)(\mathbf{x})|^2 \tag{13}$$

with $\mathcal{F}$ denoting the two-dimensional Fourier transform and $\mathcal{F}^{-1}$ its inverse. Simulations of apoferritin in this paper use Supplementary Equation (12) to form images from the exit wave ψ_e. The contribution of inelastically-scattered electrons is neglected throughout, i.e. use of an energy filter is assumed. To leading order in ψ_s (i.e. in the weak scattering case), we have

$$|\psi_i(\mathbf{x})|^2 = |H(\mathbf{0})|^2 + H(\mathbf{0}) \cdot \left(\psi_s * \mathcal{F}^{-1}[H]\right)^*(\mathbf{x}) + H^*(\mathbf{0}) \cdot \left(\psi_s * \mathcal{F}^{-1}[H]\right)(\mathbf{x}) \tag{14}$$

with $(\cdot)^*$ denoting complex conjugation. The Fourier transform of the image is then

$$\mathcal{F}[|\psi_i|^2](\mathbf{s}) = |H(\mathbf{0})|^2\delta(\mathbf{s}) + \Psi_s^*(-\mathbf{s})H(\mathbf{0})H^*(-\mathbf{s}) + \Psi_s(\mathbf{s})H^*(\mathbf{0})H(\mathbf{s}), \tag{15}$$

with $\Psi_s := \mathcal{F}[\psi_s]$. In the weak scattering case, $\psi_s(\mathbf{x}) \approx i\varphi(\mathbf{x}) - \mu(\mathbf{x})$ with $\varphi(\mathbf{x}), \mu(\mathbf{x}) \in \mathbb{R}$, so

$$\Psi_s^*(-\mathbf{s}) = -i\Phi^*(-\mathbf{s}) - M^*(-\mathbf{s}) = -i\Phi(\mathbf{s}) - M(\mathbf{s}) \tag{16}$$

where $\Phi := \mathcal{F}[\varphi]$ and $M := \mathcal{F}[\mu]$. We can then write

$$\mathcal{F}[|\psi_i|^2](\mathbf{s}) = |H(\mathbf{0})|^2\delta(\mathbf{s}) + 2\Phi(\mathbf{s})\left\{\frac{i}{2}\left[H^*(\mathbf{0})H(\mathbf{s}) - H^*(-\mathbf{s})H(\mathbf{0})\right]\right\}$$
$$- 2M(\mathbf{s})\left\{\frac{1}{2}\left[H^*(\mathbf{0})H(\mathbf{s}) + H^*(-\mathbf{s})H(\mathbf{0})\right]\right\} \tag{17}$$

The term in the braces which multiplies Φ is the (phase) contrast transfer function. To further specialize and recover main text Equation (5), we make some common assumptions.

First, we assume that the transfer function is symmetric to inversion, $H(\mathbf{s}) = H(-\mathbf{s})$. Then Supplementary Equation (17) becomes

$$\mathcal{F}[|\psi_i|^2](\mathbf{s}) = E^2(\mathbf{0})\delta(\mathbf{s}) + 2\Phi(\mathbf{s})E(\mathbf{0})E(\mathbf{s})\sin\left[\chi(\mathbf{s}) - \chi(\mathbf{0})\right] - 2M(\mathbf{s})E(\mathbf{0})E(\mathbf{s})\cos\left[\chi(\mathbf{s}) - \chi(\mathbf{0})\right]. \tag{18}$$

Second, we make the approximation, widely adopted in cryo-EM, that amplitude modulation of the scattered wave is proportional to its phase modulation, $\mu = \kappa\varphi$ for $\kappa \ll 1$ such that

$$\mathcal{F}[|\psi_i|^2](\mathbf{s}) = E^2(\mathbf{0})\delta(\mathbf{s}) + 2\Phi(\mathbf{s})E(\mathbf{0})E(\mathbf{s})\left\{\sin\left[\chi(\mathbf{s}) - \chi(\mathbf{0})\right] - \kappa\cos\left[\chi(\mathbf{s}) - \chi(\mathbf{0})\right]\right\} \tag{19}$$

$$\approx E^2(\mathbf{0})\delta(\mathbf{s}) + 2\Phi(\mathbf{s})E(\mathbf{0})E(\mathbf{s})\sin\left[\chi(\mathbf{s}) - \chi(\mathbf{0}) - \kappa\right]. \tag{20}$$

With the straightforward assumption that $E(\mathbf{0}) = 1$, we arrive at

$$\mathcal{F}[|\psi_i|^2](\mathbf{s}) = \delta(\mathbf{s}) + 2\Phi(\mathbf{s}) \cdot \mathrm{CTF}(\mathbf{s}), \tag{21}$$

$$\mathrm{CTF}(\mathbf{s}) := E(\mathbf{s})\sin\left[\chi(\mathbf{s}) - \chi(\mathbf{0}) - \kappa\right]. \tag{22}$$

In the absence of a phase plate, $\chi(\mathbf{0}) = 0$ is often set by convention and, as such, that term is sometimes not made explicit elsewhere in the literature. However, this expression illustrates that phase contrast is determined by the phase which is accumulated in relation to the unscattered wave at the origin of the diffraction plane, $\mathbf{s} = \mathbf{0}$.

Supplementary References

[1] Axelrod, J. J. *et al.* Observation of the relativistic reversal of the ponderomotive potential. *Phys. Rev. Lett.* **124**, 174801 (2020).
