# Supplementary material for: Crossed laser phase plates for transmission electron microscopy
Source: Nat Commun. 2026 Jun 5;17:7199. doi: 10.1038/s41467-026-74060-6 (PMC13396383; doi:10.1038/s41467-026-74060-6)
Supplement: Supplementary file 2 — Transparent Peer Review file [file 41467_2026_74060_MOESM2_ESM.pdf]

nature portfolio

Peer Review File

Crossed laser phase plates for transmission electron microscopy

Corresponding Author: Professor Holger Mueller

This file contains all reviewer reports in order by version, followed by all author rebuttals in order by version.

Version 0:

Reviewer comments:

Reviewer #1

(Remarks to the Author)
The authors propose a modification of laser phase plate to address several drawbacks of the single-laser beam phase plate. They perform simulations using a weak phase plate approximation. While pleasant reading, the manuscript has many severe weaknesses. Therefore, the paper should be rejected.

The benefits mostly arise from the use of two separate cavities with diffraction plane at each of them. But the abstract, Figure 1 and Introduction are misleading in believing that single cavity with two beams can be used, a scenario applicable only below 100 keV electron energy. The use of two cavities implies significant modification of the microscope column and need for simultaneous alignment at two diffraction planes. Figure 1 needs to be augumented to show two cavities and show the polarization of light in either single cavity with two laser beams and in two cavities mode. This will clarify what is the discussed scheme in the text.

As the authors point out, LPP has a conceptual problems arising from lack of radial symmetry of the laser field at the diffraction plane affecting the image and induces ghost images. Adding 2nd laser beam addresses this fundamental problem to some extent, but alternative methods (such as Zach phase plate) achieve the similar results, are easy to implement and convenient to control the phase shift. Therefore, presenting the LPP and non-existent XLPP as an unmatched solution to challenges in imaging of small molecules is incorrect.

More generally, any phase plate method published thus far does NOT improve transfer of high spatial frequencies, as required for higher resolution imaging and for imaging of small (bio) objects. Aberration correction, high brightness electron source and improved detection schemes are required for such goals. Phase plates do help with e.g. alignment of multiple images by the virtue of improving transfer of low spatial frequencies which, in some cases, can improve data resolution by the virtue of improved SNR in multiple, better aligned images of an object. This point is ignored by the authors.

As is, the paper is yet another of "tour de force" results on LPP, presented as if there was imminent and significant benefit in "ready to implement" XLPP. But in fact, the authors only have simulations (possibly incorrect due to use of weak phase object approximation).

Should the authors be able to show that their, WPO-based simulations are correct, the paper is still misleading in implying that any PP can address the high resolution imaging of small molecules, and that XLLP is ready for practical implementation. The LPP concept has been around for nearly a decade with little practical outcomes.

Nature. 2020 November 01; 587(7832): 152–156 provides some insights in the direction towards high resolution imaging of small molecules. The authors should cite this work and carefuly review their claims against the direction indicated in that paper.

If resubmitted, use line numbering in your manuscript. (in TeX use \linenumbers command).

--- specific concerns ----

The first sentence is factually incorrect:
" A phase plate increases the contrast of weak-phaseobjects in transmission electron microscopy (TEM)by phase-shifting the unscattered component of thetransmitted electron wave [1]."

To achieve contrast enhancement by phase plate techniques (see e.g. optics texts by Zernike), a RELATIVE phase shift between the uncscattered and scattered wave. There is no need for a phase shift of either direct or scattered or both components, as long as a RELATIVE phase shift between the direct and diffracted beams is achieved.

The amount of the relative phase shift between the direct and diffracted beam that provides highest contrast transfer is affected by the distribution of the spatial frequencies in the object.

Reference [1] does not discuss principles of phase plates in general. Rather it focuses on a particular type of phase plate developed by the authors. It is inadequate as it does not address up the points mentioned earlier, nor does it provide insights in concepts in phase plate imaging. The authors should make use of existing review papers providing detailed insights into phase plates and their operating principles, e.g. Microscopy 70 (1), 75-115 or earlier papers. It also provides reference to phase shift normalization applicable to weak phase object use - therefore critical reference for this paper (Micron 100,10–22)

The authors need to discuss the concepts behind the horizontal and vertical polarization of laser field clearly. They need to decide whether they discuss two beams in one cavity or two cavities configuration throughout the manuscript. Alternatively, they can separate the two configurations clearly in the manuscript. But in the current form the text is misleading: it brings up benefits of the second configuration, while significant part of the manuscript is on the first configuration.

Statement:
"... affords precise control of the electron beam phase across multiple exposures enables advanced imaging schemes ..."
is a highly misleading.

Neither the LPP not XLPP has so far demonstrated a practical "precise control of electron beam phase". As a matter of fact, the LPP results published so far are "tour the force experiments" that may be extremely difficult to implement in the cryo-TEM community. Some of the reasons why LPP is unlikely to gain track in the community are mentioned by the authors themselves in the last paragraph on p.1. The weaknesses of SLPP are somewhat addressed by the use of two lasers in two separate cavities, if the authors are correct.

In addition to fundamental issues applicable to all phase plates known to date, in terms of improving practical imaging or small molecules, the SLPP and XLPP bring further implementation difficulties, non-radial symmetry in back focal plane and ghost images.

The authors claim demonstration of LPP to cryo ET but refer to "unpublished results".
Such statements do not belong to archival scientific literature. Either publish the results, or retract the statement.

The improved imaging properties of the XLPP will add considerable value for imaging larger macromolecules and large cellular
features such as elements of subcellular ultrastructure

This statement is speculative and not supported by results. It has to be either supported or retracted.

"Stronger focusing of the LPP will allow Cs and Cc of a phase-plate TEM to be reduced without increasing the cut-on frequency, making the benefits of the LPP more accessible without compensation by advanced, expensive TEM hardware."
This statement is speculative and not supported by results. It has to be either supported or retracted.

Earlier, the authors state benefit from splitting the required laser power between two cavities, yet their Eq. (1) and (2) applies to cross lasers in one cavity. The discussion of the Eq. (1) and (2) they again seem to imply that the crossed laser beams act in one cavity, as does Figure 1.

While there could be a benefit from dividing the laser power between two cavities, it is likely to significantly increase the problems with beam drift (beam tilt), alignment of electron beam relative to laser field, requires additional diffraction plane with magnification between the two diffraction planes by factor of 1, thus requiring additional hardware. Essentially, one has to achieve a good alignment at two diffraction planes simultaneously. Therefore I find the above statement on benefit of two cavities extremely speculative and misleading.

The drawbacks of the two cavity set up need to be discussed. Fig. 1 needs to be modified to actually show the two separate cavity set up that the authors claim is benefitial.

The fact that the crossed beams laser beams in one cavity are applicable to sub 100 keV electron beam need to be emphasized, preferably right in the abstract. The abstract and Fig. 1 lead to an impression that a single cavity with two laser beams are the objective of the paper. In fact, the benefits applicable to usual 200 to 300 keV electron beam require two separate cavities with electron optical magnification = 1 adding to column complexity.

Fig 1 c) and b) are pretty, but not informative. A profile of the laser-induced phase shift should be plotted toegther with a scattering profile of a representative object, such as scattering factor f(q) a carbon atom, or a small cabon sphere. The plot of the reciprocal representation of an object and the phase shift profile allow readers to estimate the effect of the LPP / XLPP on transfer of spatial frequencies contained in an object. The cross sections through c) should be taken at a horizontal line and at 45 degree line to account for the lack of radial symmetry in LPP / XLPP. Similarly, Fig. 1d) need to include a spatial frequency representation of a test object, such as carbon atom f(q).

Fig. 1d) compares XLLP and LLP, suggesting only small benefit from implementing XLLP. Furthermore, the figure must include example transfer function from an ideal PP, or hole-free (Volta) PP that is now readily available on commercial instruments.

--- Paragraph "Contrast transfer function" ---
I'm not conviced that weak phase object (WPO) is justified. As a matter of fact, WPO implies assumption on setting the phase shift of the direct beam to 0, as discussed in Microscopy 70 (1), 75-115 and Micron 100, 10–22 Appendix A. When chosing WPO, one implicitly choses a reference point of phase plate to be 0 at zero spatial frequency. This is in disagreement of the phase plate reference point used by the authors (check Micron 100, 10–22 Appendix A for detailed analysis).

But in the first sentence of the paper, the authors state that the LPP:
"A phase plate increases the contrast of weak-phas eobjects in transmission electron microscopy (TEM)by phase-shifting the UNSCATTERED component of thetransmitted electron wave"

Which immediatelly suggests that the weak phase object approximation should not be used. The authors must show by rigorous math why WPO is applicable given its implicit setting of phase shift at zero spatial frequency to zero radians or correct their statements and simulations.

Why is the contribution of amplitude contrast not function of spatial frequency ? Such assumption is not physical.

"The more closely the phase shift ⌘ can approximate an ideal Zernike phase plate, which phase-shifts only the unscattered beam at the origin of the diffrac-
tion plane ..."
This statement is wrong. ZPP has a hole at q = 0, thus it does not affect the phase of the unscattered beam. It affects the phase shift of the scattered beams. That said, for a strong phase object one perhaps has a freedom of choice of reference point for the phase shift of a phase plate. But the authors forego this freedom by using WPO.

" The first cut-on frequency is defined as the spatial frequency at which the azimuthal average of |CTF(s)| first reaches 0.5."
... " |s| > s1 .
A second cut-on frequency of the LPP is defined by the spatial frequency at which the azimuthal average of |CTF(s)| first reaches 0.8. corresponds to the spatial frequency ... "
Taking an azimuthal average on a phase plate that does not have radial symmetry is misleading. The lack of circular symmetry induces artifacts in the imaging (which can be sometimes reduced by processing). In a paper on properties of PP (LPP and XLPP included), the potential imaging artifacts should not be purposely neglected by azimuthal averaging.

" In the XLPP, interference of the two laser beams prevents total elimination of the ghost images ... "
This yet another inconsistency discussing XLPP with two lasers at the same plane, and two lasers at two cavities (as per earlier discussion of advanatages of XLPP).

Fig. 3 needs a scale bar or state field of view for the two upper rows in the figure caption. Labels a), b) and c) are not indicated in teh figure. Meaning of PSD is not expalined in the text. The figure (presumably 3c) should include reciprocal representation of the object in (presumably) a).

--- Discussion ---
Discussion seems to provide some fair insights in the practical difficulties encountered with the LPP / XLPP.

p. 8 "" proposed a simple acquisition scheme which further suppresses ghosts,achieving a dramatic reduction of their intensity incomposite images."
Statement is misleading and applicable only when objects in the sample are sufficiently separated not to overlap with the ghost images. The proposed method is perhaps conceptually simple, but appears to be extremely difficult to implement in a practical instrument, as witnessed by the decade of LPP struggles.

The authors need to discuss the effect of inelastic scattering in a representative sample, and resulting decohernce of the

electrons, and contrast delocalization due to e.g. chromatic aberration.

The authors should, at least briefly, compare their XLPP / LPP with differential contrast imaging applied to biological materials. DPC appears to be gaining popularity, is simple to implement and addresses many of the problems that were supposed to be addressed by phase plates (of any kind).

"In addition to advancing the capabilities of phase contrast cryo-EM as characterized in this paper, development of the XLPP will broaden the parameter
space of phase plates available to electron microscopy, enabling advanced imaging schemes as ..."

This statement is speculative and should be removed. As pointed earlier (and implied by authors in the Discusion section) the interaction of the laser with high energy electrons in free space is very weak, requiring very high laser power and thus of little practical use.

---- Suppl. information -----
Vertical / horizontal polarization: how does the \theta_{1,2} relate to the direction of the electron beam ?

S2, S3 needs scale bars on plots, or at least mention field of view in 1/Angstrom in the figure caption.

S4 needs scale bars or mention of field of view or object size.

S5, S6 need a plot of reciprocal representation of a test object, such as carbon atom f(q) or a reciprocal representation of a bio molecule.

S6 ... when compared to e.g. Hole-free (aka Volta) and Zach PP, the XLPP has the disadvantage of oscillations at low spatial frequencies. This must be mentioned and discussed by the authors in the main text.

(Remarks on code availability)
I did not feel it is worth reviewing code given the high likelihood that the simulation is based on wrong assumptions arising from the use of weak phase object approximation.

Reviewer #2

(Remarks to the Author)
This paper describes, as a proof of concept study a new design for a laser phase plate based on two orthogonal polarized lasers. The essential physics underpinning this design are provided and it is critically critically compared using simulations to the authors previously published laser phase plate based on a single optical standing wave. The reduction in the “ghost” images and a potential increase in the numerical aperture may turn out to be particularly significant in this design.
My concern is that this may be too early to be suitable for Nature Comm. The paper describes a concept but there is no experimental or engineering data to show that it can be constructed and that the [performance matches the theoretical predictions. However I realise that the suitability is probably best left to the Editor to decide. If it is not suitable for Nature Comm. It would be of interest to the EM community as a novel idea and might be published in a specialist journal.

I have a small number of technical queries in addition to the comments above:
P5 The authors describe the ghost images as “fain” but this could be quantifies e.g. with reference to the primary image and wrt typical SNR in cryo EM images at fluence typically used.

P7 The contrast of the first order ghosts is decreased by roughly 2-3 X. Can the authors be more specific here as the mathematics I believe suggests a reduction of 2X if both orthogonal standing waves are identical ?
P7 It would be helpful to further expand the statement about the use of the XLPP (and other phase plates) with regards to schemes that recover the complex exit wave, as has been demonstrated at low fluence for cryo EM specimens.

Finally I would like to have seen some initial “engineering” estimates for some of the key parameters that can be realistically achieved. The authors discuss the materials properties for the mirror in brief the mirror tolerances required but I could not find any information on the laser stability or on the achievable laser focal width.

(Remarks on code availability)

Version 1:

Reviewer comments:

Reviewer #1

(Remarks to the Author)

The authors addressed my concerns, improved the clarity of the manuscript. In my opinion, the manuscript is now acceptable for publication.

(Remarks on code availability)

Reviewer #2

(Remarks to the Author)
Referee 2
I thank the authors for their comprehensive responses to my comments on the earlier version of this paper, the modifications to which have, in my view significantly improved the manuscript. My comments below refer only to their response to my original review and not to the comments of, and responses to the other referee.
Most importantly the authors now include an initial experimental demonstration of the overlapped laser beams in a shadow image which clearly demonstrates that the design concept can be experimentally realised. They have also clarified the respective signal powers in the main images and ghosts as requested. The new SI Note 5 also sets out the relevant mathematics for the reduction in the “ghost signal”. Finally the additional text and references which compare this approach to complex wavefunction recovery and suggests strategies for using a LPP in such a scheme are very helpful as are the “engineering estimates” for the laser required which are now provided.

(Remarks on code availability)

Open Access This Peer Review File is licensed under a Creative Commons Attribution 4.0 International License, which permits use, sharing, adaptation, distribution and reproduction in any medium or format, as long as you give appropriate credit to the original author(s) and the source, provide a link to the Creative Commons license, and indicate if changes were made.
In cases where reviewers are anonymous, credit should be given to 'Anonymous Referee' and the source.
The images or other third party material in this Peer Review File are included in the article’s Creative Commons license, unless indicated otherwise in a credit line to the material. If material is not included in the article’s Creative Commons license and your intended use is not permitted by statutory regulation or exceeds the permitted use, you will need to obtain permission directly from the copyright holder.
To view a copy of this license, visit https://creativecommons.org/licenses/by/4.0/

Response to Reviewers

Below, we have responded to the comments from both reviewers. *Reviewer comments are italicized*, our responses are colored blue, transcribed portions of the paper text are red, and modifications to the text are red and highlighted in yellow. Reference numbers refer to the updated manuscript.

Response to Reviewer #1

The authors propose a modification of laser phase plate to address several drawbacks of the single-laser beam phase plate. They perform simulations using a weak phase plate approximation. While pleasant reading, the manuscript has many severe weaknesses. Therefore, the paper should be rejected.

We thank the reviewer for their feedback and will clarify several misunderstandings about our work. We hope that the reviewer will find our responses satisfactory and the revised manuscript clearer.

The benefits mostly arise from the use of two separate cavities with diffraction plane at each of them. But the abstract, Figure 1 and Introduction are misleading in believing that single cavity with two beams can be used, a scenario applicable only below 100 keV electron energy.

Fig. 1a (original reproduced on the right for convenience) and the Introduction are clear: two cavities, with one beam each, form the crossed laser phase plate ("XLPP"). They operate in a single plane of the microscope. This configuration is assumed throughout the paper. We have amended the first mention of the XLPP in the Introduction to make these central concepts more obvious:

Here, we show that combining two LPPs in a single diffraction plane at 90° to each other in an "X"-shaped configuration (XLPP, shown schematically in Figure 1a) can…

We hope that the prototype cavity shown in Figure 5 further clarifies this.

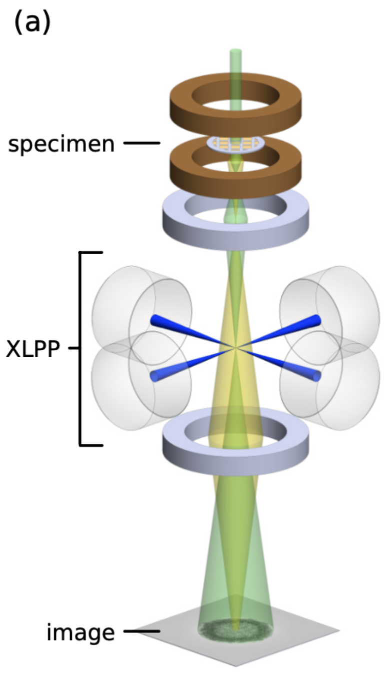

In Supplementary Notes 1 and 2, we study the effect of laser polarization on the phase shift at various electron energies. However, the geometry illustrated in Fig. 1a is assumed throughout, independent of electron energy or polarization.

The use of two cavities implies significant modification of the microscope column and need for simultaneous alignment at two diffraction planes.

We thank the reviewer for this commentary. However, this perspective overlooks that in our design the two laser beams operate in the same diffraction plane, either perfectly overlapping or within ~100 µm of each other (in the case of our current experimental prototype in Fig. 5), which is negligible for the mechanical structure of the column. Alignment of each laser to the electron beam is possible using the same methods as the alignment of the single LPP, which has already been automated (see Ref. 24). We have added further clarification in our description of the prototype cavity:
Although the cavities share a mount, using two flexures and two laser systems allows the cavities to be stabilized independently.

Figure 1 needs to be augumented to show two cavities and show the polarization of light in either single cavity with two laser beams and in two cavities mode. This will clarify what is the discussed scheme in the text.

We thank the reviewer and have augmented Fig. 1a to show the horizontal polarizations used throughout the main text and have added a clarifying sentence to draw attention to this:
…the most favorable phase plate is obtained from two non-interfering, horizontally-polarized laser beams. This is the special case, illustrated in Figure 1a, which we will consider throughout the main text of this paper.

As the authors point out, LPP has a conceptual problems arising from lack of radial symmetry of the laser field at the diffraction plane affecting the image and induces ghost images. Adding 2nd laser beam addresses this fundamental problem to some extent, but alternative methods (such as Zach phase plate) achieve the similar results, are easy to implement and convenient to control the phase shift. Therefore, presenting the LPP and non-existent XLPP as an unmatched solution to challenges in imaging of small molecules is incorrect.

This perspective is wide off the mark on several counts:

1. Overcoming the remaining technical issues mentioned by the reviewer is the central topic of our paper. The XLPP reduces the consequences of asymmetry by reducing ghost artifacts (see "Ghost suppression," page 7) and improving the laser focussing (see "Numerical aperture increase," page 5), and thereby leads to a further improvement in the cut-on spatial frequency and ghost artifacts, as illustrated in Fig. 3.
2. The LPP does not show the signal loss found in the Zach or Volta phase plates. For example, the Zach phase plate produces artifacts due to partial occlusion of the beam [Obermair et al., *Ultramicroscopy* 218 (2020)], while the Volta phase plate shows signal losses of ~18% [Danev et al., *PNAS* 111 (2014)], poor phase shift stability [Danev et al., *eLife* 5 (2016)], as well as an additional attenuation of high-resolution signal [Buijsse et al., *Ultramicroscopy* 218 (2020)]. This resolution loss was proposed as the reason why single-particle analysis with the VPP performed worse than without it in Danev et al.,

Nat. Commun. 12 (2021). We have added a discussion of alternative methods to the Introduction along with citations to these publications.

3. The XLPP is not non-existent; see below.

More generally, any phase plate method published thus far does NOT improve transfer of high spatial frequencies, as required for higher resolution imaging and for imaging of small (bio) objects. Aberration correction, high brightness electron source and improved detection schemes are required for such goals. Phase plates do help with e.g. alignment of multiple images by the virtue of improving transfer of low spatial frequencies which, in some cases, can improve data resolution by the virtue of improved SNR in multiple, better aligned images of an object. This point is ignored by the authors.

We thank the reviewer for sharing their perspective. However, phase plates can improve the transfer of high spatial frequencies as they enable in-focus imaging, and the resulting lack of CTF oscillations at high spatial frequencies doubles the overall spectral power relative to imaging with defocus (Figs. 1d and S6). Since this is similar between the SLPP and XLPP, it is not a focus of this paper, but we have added a brief clarification and a reference:
We emphasize that the improvement comes from removing CTF oscillations and not from enhancing the coherence envelope [Fan et al., *Structure* 25 (2017)].

We agree that phase plates help with alignment of images and added a mention of this to the Introduction (first paragraph) along with a reference [Zhang et al., *J. Chem. Inf. Model.* 60 (2020)].

As is, the paper is yet another of "tour de force" results on LPP, presented as if there was imminent and significant benefit in "ready to implement" XLPP. But in fact, the authors only have simulations (possibly incorrect due to use of weak phase object approximation).

We appreciate the reviewer's perspective, and have now complemented the simulations in our paper with the prototype XLPP and experimental Ronchigram demonstration in Figure 5, along with a section "*Prototype crossed laser phase plate*."

None of our simulations use the weak phase object approximation. They use the microscope transfer function *H* directly, and images are calculated using Eq. S12. The contrast transfer function (CTF) is only used schematically because it is relevant to the imaging of weakly scattering objects. To clarify the accuracy and relevance of the weak phase approximation as we have used it, we have added Supplementary Note 5, which provides a derivation of the CTF in detail and explicitly states that simulations of apoferritin in the paper use *H* rather than the CTF (Equation S12).

Should the authors be able to show that their, WPO-based simulations are correct, the paper is still misleading in implying that any PP can address the high resolution imaging of small molecules, and that XLLP is ready for practical implementation. The LPP concept has been around for nearly a decade with little practical outcomes.

This commentary is off the mark on several counts. First, phase plates are widely expected by the community to improve reconstruction of small particles (e.g. Refs. 4, 23, 33), not just by us, and this has been corroborated by simulations (e.g. in Ref. 5). Second, as will be addressed more fully later in this response, our simulations do not use the weak phase approximation (see also Supplementary Note 5). Regarding "practical outcomes" of the LPP, we refer the reviewer to Refs. 18, 21, and 24 for examples of substantial experimental progress from 2021 to present. We have drawn attention to these practical outcomes in the Introduction:
The LPP has demonstrated the full π/2 rad phase shift desired for imaging weak-phase objects [18, 19], as well as stable and analytically-tractable properties over the time needed to take large datasets [20, 21]. These features of the LPP have made it a leading candidate to replace previous phase plate designs in cryo-EM [22, 23]. Application of the LPP to single-particle analysis has already been demonstrated [18, 24].

Nature. 2020 November 01; 587(7832): 152–156 provides some insights in the direction towards high resolution imaging of small molecules. The authors should cite this work and carefuly review their claims against the direction indicated in that paper.

Thanks for the reference. However, we must point out that while its authors do not mention phase plates, the hardware developments they describe (improved electron source and detector) are compatible with phase plates. We also point out *"Cryomicroscopy in situ: what is the smallest molecule that can be directly identified without labels in a cell?"* (2022, Ref. 23 in the updated manuscript and 14 in the original), which highlights a LPP as a candidate to improve the detection limit of cryo-EM alongside liquid helium cooling and chromatic aberration correction. We do not claim that phase plates are the only means to improving imaging of small proteins; on the contrary, our paper states:
…improvement of the LPP design directly is preferred so that other microscope hardware and software can be used to its full advantage.

If resubmitted, use line numbering in your manuscript. (in TeX use \linenumbers command).

We have done so in the updated manuscript.

--- specific concerns ----

The first sentence is factually incorrect:
" A phase plate increases the contrast of weak-phaseobjects in transmission electron microscopy (TEM)by phase-shifting the unscattered component of thetransmitted electron wave [1]."

To achieve contrast enhancement by phase plate techniques (see e.g. optics texts by Zernike), a RELATIVE phase shift between the uncscattered and scattered wave. There is no need for a phase shift of either direct or scattered or both components, as long as a RELATIVE phase shift between the direct and diffracted beams is achieved.

We appreciate the concern about clarity. This fact was already obvious from Eq. 5 in the previous version, but we have amended the sentence to
phase-shifting the unscattered component of the transmitted electron wave relative to the scattered component
and added a remark in Supplementary Note 5 (in which we have derived Eq. 5):
phase contrast is determined by the phase which is accumulated in relation to the unscattered wave at the origin of the diffraction plane.

The amount of the relative phase shift between the direct and diffracted beam that provides highest contrast transfer is affected by the distribution of the spatial frequencies in the object.

We agree with the reviewer, but this observation is not specific to the XLPP and thus beyond the scope of this work. We chose to focus on the CTF in order to maintain the generality of this work. The amount of phase shift that maximizes the CTF in Eq. 5 is as presented in the text.

We have added a statement to the Introduction which clarifies the context in which 90 degrees of total phase shift is sought and provides a reference to a paper which elaborates on object-dependence of this optimum [Danev & Nagayama, *Ultramicroscopy* 111 (2011)]:
The LPP has demonstrated the full π/2 rad phase shift desired for imaging weak-phase objects [18, 19],

Reference [1] does not discuss principles of phase plates in general. Rather it focuses on a particular type of phase plate developed by the authors. It is inadequate as it does not address up the points mentioned earlier, nor does it provide insights in concepts in phase plate imaging. The authors should make use of existing review papers providing detailed insights into phase plates and their operating principles, e.g. Microscopy 70 (1), 75-115 or earlier papers. It also provides reference to phase shift normalization applicable to weak phase object use - therefore critical reference for this paper (Micron 100,10–22)

This remark overlooks that Ref. 1 devotes much discussion to the principles of phase plates in general and alternative approaches. Indeed, 39 out of 45 references cited therein are about these and not about the laser phase plate. Citing this paper adds key recent references on Volta phase plates not found in the older review suggested by the reviewer such as Buijsse et al., *Ultramicroscopy* 218 (2020), Danev et al., *Nat. Commun.* (2021), which are motivating the further development of the LPP. We have added citations to these papers. To additionally provide a historical perspective, we have added citations to Glaeser, *Rev. Sci. Instrum.* 11 (2013) and, per the reviewer's suggestion, Malac et al., *Microscopy* 70 (2021).

Phase shift "normalization" was discussed in Ref. 17 and is freshly derived for readers in Supplementary Note 5. It was also present in Eq. 5 in the original manuscript.

The authors need to discuss the concepts behind the horizontal and vertical polarization of laser field clearly. They need to decide whether they discuss two beams in one cavity or two cavities

configuration throughout the manuscript. Alternatively, they can separate the two configurations clearly in the manuscript. But in the current form the text is misleading: it brings up benefits of the second configuration, while significant part of the manuscript is on the first configuration.

We reiterate that the configuration presented in Fig. 1a is the only one considered. The manuscript already devotes much room to the effects of horizontal and vertical polarization. Eq. 1 is valid for arbitrary linear polarizations. Whether the beams should be horizontally- or vertically-polarized is addressed in the Discussion, with detailed derivations presented in Supplementary Notes 1 and 2.

To make this more clear, as mentioned above we have added a remark to the beginning of the Results section:
…at high accelerating voltages the most favorable phase plate is obtained from two non-interfering, horizontally-polarized laser beams. This is the special case, illustrated in Figure 1a, which we will consider throughout the main text of this paper.
and to the Discussion section
In this important respect, choosing to operate the XLPP using horizontally-polarized laser beams simplifies these design constraints …

Statement:
"... affords precise control of the electron beam phase across multiple exposures enables advanced imaging schemes ..."
is a highly misleading.

Neither the LPP not XLPP has so far demonstrated a practical "precise control of electron beam phase". As a matter of fact, the LPP results published so far are "tour the force experiments" that may be extremely difficult to implement in the cryo-TEM community. Some of the reasons why LPP is unlikely to gain track in the community are mentioned by the authors themselves in the last paragraph on p.1. The weaknesses of SLPP are somewhat addressed by the use of two lasers in two separate cavities, if the authors are correct.

We respectfully disagree with the reviewer on this point. The (S)LPP has indeed demonstrated practical precise control of electron beam phase.

- Ref. 17 shows precise experimental control, in agreement with a quantitative model of image formation; Ref. 20 expands upon voltage- and polarization-dependence of the laser-electron interaction and shows excellent agreement between theory and experiments.
- Ref. 24 demonstrates stability of the SLPP phase shift of 90±10 deg over hours, which far outpaces the stability of the Volta phase plate. It also demonstrates single-particle reconstructions using the SLPP.
- The interest in the broader community is illustrated by various recent works, such as Refs. 22 and 23.

The remaining weaknesses of the SLPP (page 2) motivate the simulations and experimental development of the XLPP presented here.

In addition to fundamental issues applicable to all phase plates known to date, in terms of improving practical imaging or small molecules, the SLPP and XLPP bring further implementation difficulties, non-radial symmetry in back focal plane and ghost images.

Indeed, these are the core issues addressed in our paper. Figs. 3 and 4 demonstrate how ghost images are suppressed in various ways using the XLPP, while the Discussion presents details of a potential experimental implementation, based on the successful implementation of the SLPP.

On the difficulty of implementation, we remind the reviewer that microscopes with the needed modifications have already been set up at Berkeley and Biohub (formerly the Chan-Zuckerberg Imaging Institute).

The authors claim demonstration of LPP to cryo ET but refer to "unpublished results".
Such statements do not belong to archival scientific literature. Either publish the results, or retract the statement.

We have removed this statement, as it indeed has little bearing on the current work. These results with the LPP have been presented at conferences and will be published in the literature at a later date.

The improved imaging properties of the XLPP will add considerable value for imaging larger macromolecules and large cellular
features such as elements of subcellular ultrastructure

This statement is speculative and not supported by results. It has to be either supported or retracted.

This statement by the reviewer overlooks the ample support in our manuscript for the notion that the improved properties of the XLPP will be valuable for imaging macromolecules. For example, increased information at low spatial frequencies, as shown in Figs. 1d and 2, improves imaging of large structures (Mahamid et al., *Science* 531 (2016), Ref. 7). We have slightly modified the text and added another citation to this paper to support this statement.

"Stronger focusing of the LPP will allow Cs and Cc of a phase-plate TEM to be reduced without increasing the cut-on frequency, making the benefits of the LPP more accessible without compensation by advanced, expensive TEM hardware."

This statement is speculative and not supported by results. It has to be either supported or retracted.

We thank the reviewer for the opportunity to clarify, but this is a straightforward conclusion: Stronger focussing of the laser beam allows obtaining the same cut-on spatial frequency with a smaller effective focal length of the microscope. This, in turn, decreases *Cs* and *Cc* (e.g., Grivet 1972, *Electron Optics*, p. 198), which would otherwise have to be overcome by advanced and expensive approaches, like monochromation or *Cs* correction. To clarify this, we have amended the Discussion (page 9) with an explanation of this point.
Equations (7) and (8) illustrate that an increase in N_A or decrease in λ_l also enables future microscope designs to have shorter effective focal length, f, without reducing the cut-on frequency. This bears the advantage that aberration coefficients generally increase with increasing f, so the trade-off between de-magnifying the phase plate and increasing C_c and C_s should be evaluated when designing a new phase-contrast TEM instrument. The parameters chosen in this paper (Table S1) reflect the microscope configuration currently in operation at UC Berkeley.

Earlier, the authors state benefit from splitting the required laser power between two cavities, yet their Eq. (1) and (2) applies to cross lasers in one cavity. The discussion of the Eq. (1) and (2) they again seem to imply that the crossed laser beams act in one cavity, as does Figure 1.

We remind the reviewer that the manuscript always considers two cavities, so these comments are unsound. In particular, Eq. 1 describes the electric field of two intersecting laser cavities operating at the same wavelength (Fig. 1a). The degree of interference of the laser beams is determined by the choice of laser polarization, as discussed in the Supplementary Notes: If the laser polarizations are horizontal, and thus orthogonal to one another (as in e.g. Fig 1a), then the two beams do not interfere. This makes their overlap less crucial and simplifies the experimental implementation, since the beams need only to be *near* the diffraction plane, and one another. The Discussion draws attention to this point.

While there could be a benefit from dividing the laser power between two cavities, it is likely to significantly increase the problems with beam drift (beam tilt), alignment of electron beam relative to laser field, requires additional diffraction plane with magnification between the two diffraction planes by factor of 1, thus requiring additional hardware. Essentially, one has to achieve a good alignment at two diffraction planes simultaneously. Therefore I find the above statement on benefit of two cavities extremely speculative and misleading.

The drawbacks of the two cavity set up need to be discussed. Fig. 1 needs to be modified to actually show the two separate cavity set up that the authors claim is benefitial.

As already explained, Fig. 1a is correct and the manuscript is consistent. Alignment only needs to be controlled in one diffraction plane (which contains both cavities comprising the XLPP). The laser-electron alignment procedure has already been fully automated with a high degree of

accuracy and precision over many hours (see e.g. Ref. 24, Fig. 1 for two recent experimental results).

The fact that the crossed beams laser beams in one cavity are applicable to sub 100 keV electron beam need to be emphasized, preferably right in the abstract. The abstract and Fig. 1 lead to an impression that a single cavity with two laser beams are the objective of the paper. In fact, the benefits applicable to usual 200 to 300 keV electron beam require two separate cavities with electron optical magnification = 1 adding to column complexity.

This comment appears to misunderstand the results of Supplementary Note 2. Only the configuration shown in Fig. 1a is considered, for all laser polarizations and electron energies. The laser beams intersect but their degree of interference depends on their polarizations, as expressed in Eq. 1. Their effect on the electron beam further depends on the electron beam energy (Supplementary Note 1). Supplementary Note 2 shows that vertical polarization has some benefits up to ~100 keV (Fig. S3), which disappear as the energy is increased (Fig. S2). We summarize this point in the Discussion section, with a recommendation that horizontal polarizations be used, such that the fields of the two (intersecting) lasers do not interfere:
Because the horizontally- (and therefore orthogonally-) polarized beams will not interfere
As mentioned, this has the additional benefit of a lower optical power requirement for the same phase shift.

In the new section "Prototype crossed laser phase plate," detailing our experimental implementation of the XLPP, we explain that
Thermo Fisher Scientific designed several different microscope layouts in which the diffraction plane is accessible via a rectangular port sized 27 mm×51 mm. This is larger than the port available for previous SLPP work (diameter 25 mm, circular).
It is nevertheless a single port for a single device, which is shown in Fig. 5a.

Fig 1 c) and b) are pretty, but not informative. A profile of the laser-induced phase shift should be plotted toegther with a scattering profile of a representative object, such as scattering factor f(q) a carbon atom, or a small cabon sphere. The plot of the reciprocal representation of an object and the phase shift profile allow readers to estimate the effect of the LPP / XLPP on transfer of spatial frequencies contained in an object. The cross sections through c) should be taken at a horizontal line and at 45 degree line to account for the lack of radial symmetry in LPP / XLPP. Similarly, Fig. 1d) need to include a spatial frequency representation of a test object, such as carbon atom f(q).

We respectfully disagree with the reviewer. Figs. 1b and 1c illustrate the phase shift η imparted by the SLPP and XLPP on different spatial frequency components (Eq. 2), including the ones along a 45-degree axis requested by the reviewer, emphasizing the effect that the laser-electron interaction has on the optical system in the two cases. This effect is independent of the object being studied, and readers are free to overlay it with an object of interest to them, or perhaps an

analytical model [e.g. as shown in Rosenthal & Henderson, *J. Mol. Biol.* 333 (2003), Fig. 1]. We likewise believe that the azimuthally-averaged |CTF| plot is more meaningful than two line scans because in general an object will be oriented arbitrarily with respect to the laser beams, while the lack of symmetry is apparent from Figs. 1b and 1c.

Fig. 1d) compares XLLP and LLP, suggesting only small benefit from implementing XLLP. Furthermore, the figure must include example transfer function from an ideal PP, or hole-free (Volta) PP that is now readily available on commercial instruments.

While the benefit to the azimuthal average of |CTF| appears small in Fig. 1d, this figure does not show other benefits which are central to the paper's results, such as ghost suppression (Fig. 3) and the flexibility to implement multi-image acquisition schemes (such as in Fig. 4). It is also important to note that the x-axis in Fig. 1d is on a logarithmic scale.

We chose not to feature the Volta phase plate in the figure because it distracts from the message of the paper, namely comparison of the SLPP and XLPP. Additionally, since the Volta phase plate lacks a quantitative analytical model and suffers from significant signal loss and instability (Refs. 10-13), we feel this comparison is unwarranted and has already been made qualitatively elsewhere. Likewise, we feel that addition of an "ideal" phase plate profile would reduce readability, and we have chosen to limit its discussion to the sentence on page 4
The CTF of a LPP falls short of the ideal CTF of unity in two important ways…

--- Paragraph "Contrast transfer function" ---
I'm not conviced that weak phase object (WPO) is justified. As a matter of fact, WPO implies assumption on setting the phase shift of the direct beam to 0, as discussed in Microscopy 70 (1), 75-115 and Micron 100, 10–22 Appendix A. When chosing WPO, one implicitly choses a reference point of phase plate to be 0 at zero spatial frequency. This is in disagreement of the phase plate reference point used by the authors (check Micron 100, 10–22 Appendix A for detailed analysis).

But in the first sentence of the paper, the authors state that the LPP:
"A phase plate increases the contrast of weak-phas eobjects in transmission electron microscopy (TEM)by phase-shifting the UNSCATTERED component of thetransmitted electron wave"

Which immediatelly suggests that the weak phase object approximation should not be used. The authors must show by rigorous math why WPO is applicable given its implicit setting of phase shift at zero spatial frequency to zero radians or correct their statements and simulations.

We respectfully disagree with the reviewer on this point. The weak phase *object* approximation makes an approximation regarding the *object*, not the phase origin. The CTF formalism can be applied as in Eq. 5 with proper accounting for the phase origin. This is mathematically described, for example, in Ref. 17 (Eq. S24), and emphasizes that the phase shift relative to the zero-frequency component, $\chi(\mathbf{0})$, is of interest. To further clarify this point, we have derived Eq.

5 in the new Supplementary Note 5. We note that although we did not use the CTF for image simulations, it is widely used in cryo-EM in the form derived there and serves as a useful and familiar diagnostic for comparing microscope configurations for cryo-EM, which is how we use it here.

Why is the contribution of amplitude contrast not function of spatial frequency ? Such assumption is not physical.

This is a standard assumption made in cryo-EM of biological molecules (e.g. Glaeser, *Single-particle Cryo-EM of Biological Macromolecules* (2021), p. 12; Rohou & Grigorieff, *J. Struct. Biol.* 192 (2015), Eq. 4) which amounts to assuming that the amplitude modulation of the specimen is proportional to the phase modulation by a small constant. This approximation is heavily used throughout the cryo-EM literature because it is simple but much more accurate than approximating amplitude contrast as zero, particularly at low spatial frequencies. The incorporation of amplitude contrast into the CTF is derived in the new Supplementary Note 5.

*"The more closely the phase shift ⌘ can approximate an ideal Zernike phase plate, which phase-shifts only the unscattered beam at the origin of the diffrac-
tion plane ..."*
This statement is wrong. ZPP has a hole at q = 0, thus it does not affect the phase of the unscattered beam. It affects the phase shift of the scattered beams. That said, for a strong phase object one perhaps has a freedom of choice of reference point for the phase shift of a phase plate. But the authors forego this freedom by using WPO.

As the reviewer pointed out previously, it is the *relative* phase shift between the scattered and unscattered beams that is relevant. Whether an *ideal* Zernike phase plate phase-shifts only the unscattered beam or everything but the unscattered beam is irrelevant (see e.g. Zernike, *Physica* 9 (1942), p. 697). In light of that, we chose to frame the sentence in the way that is most consistent with the rest of the paper.

" The first cut-on frequency is defined as the spatial frequency at which the azimuthal average of |CTF(s)| first reaches 0.5." ... " |s| > s1 .
A second cut-on frequency of the LPP is defined by the spatial frequency at which the azimuthal average of |CTF(s)| first reaches 0.8. corresponds to the spatial frequency ... "
Taking an azimuthal average on a phase plate that does not have radial symmetry is misleading. The lack of circular symmetry induces artifacts in the imaging (which can be sometimes reduced by processing). In a paper on properties of PP (LPP and XLPP included), the potential imaging artifacts should not be purposely neglected by azimuthal averaging.

We disagree with the reviewer's characterization here in two ways:

(1) The asymmetry of the LPP is not being purposely neglected - quite the opposite. Figs. 1b and 1c clearly showcase the asymmetry and much of the paper centers around exploring how the XLPP reduces the artifacts created by asymmetry, such as by

distributing the power between two beams and enabling an increase in numerical aperture.

(2) The quoted sentences are simply definitions; they are not misleading. The azimuthally-averaged profile of |CTF| is a simple, useful one-dimensional function to enable comparison between different imaging configurations and phase plates. It is not used in the paper except to study the differences between microscope configurations. Additionally, since an object's orientation with respect to the axes defined by the laser beam is, in general, arbitrary, the azimuthal average provides important information to a user. In single-particle cryo-EM, for example, particles are imaged at many orientations, so the azimuthally-averaged |CTF| is of great interest. Meanwhile, Eqs. 7 and 8 provide approximations, showing how key parameters of the LPP and microscope affect the scaling of the two cut-on frequencies, while more complicated, quantitative expressions describing the phase shift in the diffraction plane are presented elsewhere in the paper.

" In the XLPP, interference of the two laser beams prevents total elimination of the ghost images ... "
This yet another inconsistency discussing XLPP with two lasers at the same plane, and two lasers at two cavities (as per earlier discussion of advanatages of XLPP).

As previously stated, this is not an inconsistency but a misunderstanding by the reviewer. The two laser beams are in the same plane, within two cavities whose beams intersect, causing interference to a degree which depends upon their polarizations.

In the experimental XLPP we have presented here, a single device containing two cavities (Fig. 5a) is inserted into a single diffraction plane. We clarify in the "Prototype crossed laser phase plate" section:
By design, the two beams are offset along the electron-optical axis by 100 μm to avoid possible interference effects in this first prototype. Since the laser beams are still approximately within the Rayleigh range of the unscattered beam in the diffraction plane, they appear coplanar and the CTF should be the same as predicted in this paper.
This is shown in Fig. 5b. We have restated this in the Discussion section for clarity:
mechanical alignment tolerances are relaxed compared to the interfering case. This is already observed with the prototype presented here (Figure 5b).

Fig. 3 needs a scale bar or state field of view for the two upper rows in the figure caption. Labels a), b) and c) are not indicated in teh figure. Meaning of PSD is not expalined in the text. The figure (presumably 3c) should include reciprocal representation of the object in (presumably) a).

We have added clarification of the dimensions in Figs. 3, 4, and S4 to the figure captions and added the abbreviation "PSD" in parentheses following the first mention of the power spectral density in the main text.

We thank the reviewer for catching the missing panel labels in Fig. 3. We have updated Fig. 3c with a plot of the reciprocal space power spectrum of the object. Since the contrast transfer of

all of the phase plates here is nearly optimal and the y-axis spans many orders of magnitude, this line overlaps almost perfectly with the solid lines corresponding to PSDs of main images, except for a very slight attenuation near 1/(1 Å) where the CTF envelope starts to decrease. To highlight this, we have modified the caption as follows:
PSD of the object is shown in (c) as a gray line indicated by an arrow. It closely follows the other solid lines, departing near 1/(1 Å) due to signal attenuation by the CTF envelope.

--- Discussion ---
Discussion seems to provide some fair insights in the practical difficulties encountered with the LPP / XLPP.

We are glad for the positive review of the Discussion section.

p. 8 "" proposed a simple acquisition scheme which further suppresses ghosts,achieving a dramatic reduction of their intensity incomposite images."
Statement is misleading and applicable only when objects in the sample are sufficiently separated not to overlap with the ghost images. The proposed method is perhaps conceptually simple, but appears to be extremely difficult to implement in a practical instrument, as witnessed by the decade of LPP struggles.

Experimental demonstrations (e.g. Refs. 17, 20, 24) have shown that the phase shift produced by the LPP can be robustly controlled by slight deflection of the diffraction pattern and the agreement to the theoretical values is excellent at various laser polarizations and electron energies. The alignment of the laser and electron beams using electron deflectors has already been automated (Ref. 24), and very similar ones will enable the proposed scheme with high fidelity. We thus respectfully disagree; the proposed method will not be difficult to implement.

We also wish to clarify that the objects need not be sufficiently separated to not overlap with ghost images. One way to see this is to imagine the apoferritin particle in Fig. 4 as many objects (atoms) which are closely spaced. Another way to see it is to consider that for wave functions like those in Supplementary Note 5, image formation becomes essentially convolutional in real space (e.g., consider the inverse Fourier transform of Eq. S21). The images in Fig. 4b and 4c illustrate that the convolution kernel (point spread function) retains the same sign for the main image but changes sign for the ghost images. As a result, the approach of Fig. 4 works even for extended objects. We have summarized this in the main text with an additional sentence:
We note that this two-image approach to ghost suppression also works for extended objects (larger than $2d_g$), provided that their phase and amplitude modulation of the electron wave are approximately proportional, as assumed in cryo-EM (see Supplementary Note 5).

The authors need to discuss the effect of inelastic scattering in a representative sample, and resulting decohernce of the electrons, and contrast delocalization due to e.g. chromatic aberration.

In this paper, we have neglected the contribution of inelastically-scattered electrons under the assumption that an energy filter is used. To clarify this point, we have added to Supplementary Note 5 the sentence

The contribution of inelastically-scattered electrons is neglected throughout, i.e. use of an energy filter is assumed.

Decoherence due to chromatic and spherical aberration is treated using the envelope function *E* in Eq. 5, and produces the falloff in |CTF| at high spatial frequencies (e.g. Figs. 1d, 2, S6). The aberration coefficients are provided in Table S1 and were measured experimentally in our microscope. Decoherence due to the phase plate itself has been measured to be negligible in a LPP when the bore through the cavity through which the electron beam passes is made sufficiently wide (Ref. 20). This was done in the XLPP prototype (Fig. 5a).

The authors should, at least briefly, compare their XLPP / LPP with differential contrast imaging applied to biological materials. DPC appears to be gaining popularity, is simple to implement and addresses many of the problems that were supposed to be addressed by phase plates (of any kind).

We believe that this discussion is outside the scope of this paper and more appropriate for a separate article. It is touched on, for example, in Ref. 1.

"In addition to advancing the capabilities of phase contrast cryo-EM as characterized in this paper, development of the XLPP will broaden the parameter space of phase plates available to electron microscopy, enabling advanced imaging schemes as ..."

This statement is speculative and should be removed. As pointed earlier (and implied by authors in the Discusion section) the interaction of the laser with high energy electrons in free space is very weak, requiring very high laser power and thus of little practical use.

We disagree that this statement is speculative. Electron phase optics which produce the patterns created by the XLPP do not currently exist, hence development of the XLPP will broaden the parameter space of phase plates…

The reviewer is correct that the laser-electron interaction is weak, requiring high laser powers. Indeed, reducing the laser powers needed is a priority in the conception of the XLPP and one of its advantages over the SLPP, as discussed in the paper. Nevertheless, multiple experiments since 2021 (Refs. 18, 21, 24) have demonstrated that the required phase shift has been achieved even in a SLPP and that single-particle cryo-EM of biomolecules has already been achieved, importantly without the signal loss and degraded information at high spatial frequencies that have been reported by users of the Volta phase plate, for example, and with superior phase shift stability.

---- Suppl. information -----

Vertical / horizontal polarization: how does the \theta_{1,2} relate to the direction of the electron beam ?

The manuscript states "θ_j are polarization angles relative to the $\boldsymbol{r}_z$ axis…" and the orientation with respect to the other two axes is described in Eqs. S2 and S3. For additional clarification, we have added an axis illustration to Figs. 1a and 5a.

S2, S3 needs scale bars on plots, or at least mention field of view in 1/Angstrom in the figure caption.

We have added a statement of the field of view to the figure captions. In this case microns are more appropriate than 1/Å because of the changing electron energies.

S4 needs scale bars or mention of field of view or object size.

We have added a statement of the field of view to the figure caption. The corresponding line scans also provide this information.

S5, S6 need a plot of reciprocal representation of a test object, such as carbon atom f(q) or a reciprocal representation of a bio molecule.

We respectfully disagree with the reviewer on this point. The purpose of a CTF plot is to provide a general description of the imaging system independent of specimen. We believe adding a particular object's structure factor would be distracting to reviewers who are otherwise able to choose from a wide range of available test objects of their own interest.

S6 ... when compared to e.g. Hole-free (aka Volta) and Zach PP, the XLPP has the disadvantage of oscillations at low spatial frequencies. This must be mentioned and discussed by the authors in the main text.

We thank the reviewer for this suggestion. Although we point out that the XLPP is not plotted in Fig. S6, it is indeed true that the standing wave structure of LPPs creates oscillations which, although much less severe than the high-spatial-frequency CTF oscillations produced by widely-used defocus aberration (as seen in Fig. S6), do indeed merit a comment. In fact, the ability of the XLPP to reduce CTF oscillations in the CTF is the message of Fig. S5. To draw special attention to the oscillation at low spatial frequencies, we have added this description to the main text:

Weak oscillations of $|\mathrm{CTF}(\mathbf{s})|$ in the region $s_1<|\mathbf{s}|<s_2$ are observed due to the azimuthal average featuring some spatial frequencies which are phase-shifted by the laser light.

We also stress to the reviewer that, although the Volta and Zach phase plates do not produce CTF oscillations, they suffer from their own artifacts and instabilities. For instance, the Zach phase plate simply throws out some low-spatial-frequency components by physically blocking them.

Reviewer #1 (Remarks on code availability):

I did not feel it is worth reviewing code given the high likelihood that the simulation is based on wrong assumptions arising from the use of weak phase object approximation.

Our simulations do not use the weak phase object approximation. In fact, we perform a wave optical calculation using a transfer function applied in the Fourier plane to a complex exit wave function. We clarify this in Supplementary Note 5 with the sentence
Simulations of apoferritin in this paper use Equation (S12) to form images from the exit wave ψ_e.

==

Response to Reviewer #2

This paper describes, as a proof of concept study a new design for a laser phase plate based on two orthogonal polarized lasers. The essential physics underpinning this design are provided and it is critically critically compared using simulations to the authors previously published laser phase plate based on a single optical standing wave. The reduction in the “ghost” images and a potential increase in the numerical aperture may turn out to be particularly significant in this design.

We thank the reviewer for the accurate summary and the favorable evaluation of our work.

My concern is that this may be too early to be suitable for Nature Comm. The paper describes a concept but there is no experimental or engineering data to show that it can be constructed and that the [performance matches the theoretical predictions. However I realise that the suitability is probably best left to the Editor to decide. If it is not suitable for Nature Comm. It would be of interest to the EM community as a novel idea and might be published in a specialist journal.

We appreciate the reviewer’s perspective and believe that our revision addresses this concern. We now describe a prototype XLPP, which has been built and tested in a Thermo Fisher Krios microscope (Fig. 5a). This includes a demonstration of a Ronchigram image of its overlapped laser beams (Fig. 5b), confirming the theoretical prediction of Fig. 1c. Another indication of the timeliness and impact of our work is illustrated by a recent theoretical study, published after our original paper submission, finding that crossed laser standing waves in a similar configuration to our XLPP can be used as a spherical aberration corrector. We have added this to the introduction, with a reference:
In addition, a pair of crossed laser beams with aligned intensity antinodes, in a configuration similar to the prototype XLPP presented here, can be used to correct spherical aberration of an electron microscope, as shown in a recent theoretical study [25].

I have a small number of technical queries in addition to the comments above:
P5 The authors describe the ghost images as "fain" but this could be quantifies e.g. with reference to the primary image and wrt typical SNR in cryo EM images at fluence typically used.

We have quantitatively compared the signal power of the main images and ghosts in Fig. 3c in a spatial-frequency-dependent manner. Additionally, in Fig. 4e we plot the ratio of the spectral powers. Fig. 4e shows that at spatial frequencies of ~1/(100 Å), comparable to the molecular size, the ghost images have 0.1x the spectral power in the SLPP, which is reduced to nearly 0.001x by using the XLPP.

As for comparison to typical cryo-EM (without a phase plate), while we prefer not to choose parameters as would be needed to provide a PSD curve, we have added a gray line to Fig. 3c which denotes the PSD of the object (with perfect contrast transfer). Conventional imaging with a rapidly oscillating CTF at high spatial frequencies (as in e.g. Fig. S6) captures approximately half the information at high frequencies, so the PSD would be ~2x lower than the solid lines in Fig. 3c in that region, where ghost images are 6 orders of magnitude lower.

P7 The contrast of the first order ghosts is decreased by roughly 2-3 X. Can the authors be more specific here as the mathematics I believe suggests a reduction of 2X if both orthogonal standing waves are identical ?

The reviewer has identified a somewhat subtle point. The contrast of ghosts is reasonably well-approximated by the simple theory of a one-dimensional sinusoidal phase grating. In this approximation, to lowest order, the contrast of the ghost image scales like $J_1(\eta/2)/J_0(\eta/2)$, where J_0 and J_1 are Bessel functions of the first kind and η is the phase shift of the unscattered beam (proportional to the laser intensity). We find that $2J_1(\pi/4)/J_0(\pi/4)=0.43$ corresponds to a $\pi/2$ phase shift while if the laser is at half-power $2J_1(\pi/8)/J_0(\pi/8)=0.20$, weaker by slightly more than a factor of 2. The difference grows slightly when considering amplitude contrast and higher-order terms in the phase grating theory. The factor of 2-3x is just a qualitative description of what we observe in image space when performing full wave-optical simulations of apoferritin, as in Figs. 3 and 4, using the transfer function H, as in Eq. S12 in the new Supplementary Note 5.

P7 It would be helpful to further expand the statement about the use of the XLPP (and other phase plates) with regards to schemes that recover the complex exit wave, as has been demonstrated at low fluence for cryo EM specimens.

We thank the reviewer for pointing this out. We have added added citations to several papers describing such approaches and also elaborated for clarity in the text:
Such optics like the one described here enable more sophisticated patterning of the electron phase across multiple exposures to extract different types of information about the specimen [38-40].
The most straightforward way to incorporate a phase plate into exit wave reconstruction is to acquire successive images with the phase plate 'on' and 'off', measuring the phase and then the amplitude of the electron wave (as described in Ref. 38, for example).

Finally I would like to have seen some initial "engineering" estimates for some of the key parameters that can be realistically achieved. The authors discuss the materials properties for the mirror in brief the mirror tolerances required but I could not find any information on the laser stability or on the achievable laser focal width.

The reason we focus on the mirror design in the Discussion is that it has the most direct effect on the behavior of the XLPP as a phase plate and the SLPP mirror design is already state-of-the-art in terms of surface roughness and radius of curvature. However we are happy to add information on other parameters. The laser focal width w_0 ($1/e^2$ beam waist radius) is inversely proportional to the cavity numerical aperture as stated just after Eq. 3 in the text. In the paper, we state that "...preliminary experiments suggest that twofold reduction in the circulating power should enable an increase of N_A to ~0.08 in a XLPP." This entails a beam waist of 4.2 microns, compared with the waist at N_A=0.05 of 6.7 microns in the SLPP. The laser linewidth should be smaller (e.g. 10x or more) than the cavity linewidth to enable locking using the Pound-Drever-Hall approach used by the SLPP. We have addressed this point with the following addition to the text:

First, a suitable high-power, narrow-linewidth laser with the chosen wavelength must be available for cavity locking. The SLPP cavity linewidth is ~200 kHz, so a laser with ~3 kHz free-running linewidth is used, with a total power of ~12 W at the cavity input.
